# Review of *From Darwin to Derrida* by David Haig: MIT Press 2020. ISBN 9780262043786

**DOI:** 10.1007/s10441-020-09398-5

**Published:** 2020-10-22

**Authors:** Samir Okasha

**Affiliations:** Department of Philosophy, University of Bristol, Bristol, BS6 6JL UK

David Haig is an evolutionary biologist best known for his pioneering work on the evolutionary implications of genomic imprinting, the remarkable phenomenon in which the phenotypic effect of a gene depends on whether it was maternally or paternally inherited. Haig’s key contribution was to connect genomic imprinting with the theory of kin selection, which led him to the insight that the maternally and paternally derived alleles of a gene within an organism would be selected to favour different phenotypic strategies, leading to within-organism conflict. In addition to his scientific work, for many years Haig has also been an active participant in the philosophy of biology, engaging with many of the main topics in that field. The 15 essays collected in *From Darwin to Derrida* are a reflection of Haig’s dual scientific and philosophical interests. The chapters bear intriguing titles (“Barren Virgins”, “Vive La Différance”, “Darwinian Hermeneutics”) and stem from Haig’s conviction that “the humanities and sciences have much to say to each other” (p. xxvii). Haig’s book touches on a dizzyingly broad array of topics—from the messy molecular details of riboswitches to the nature of human freedom. The book is written in a style that is informal and readable, despite tackling issues of considerable scientific and philosophical complexity.

The book’s subtitle—“Selfish Genes, Social Selves and the Meaning of Life’’—reflects some of the main themes. Haig’s thinking has been heavily influenced by the selfish gene theory of Richard Dawkins, and he has much to say about the gene concept, the power and limits of gene selectionism, the notion of replication, cultural evolution, memetics, and related topics. “Social Selves” refers to Haig’s conviction that an individual organism is a collective entity, made up of genomic constituents with non-identical evolutionary interests. Haig argues that the resulting intra-genomic conflict, for example between imprinted genes at the same locus, or between nuclear and cytoplasmic genes, is analogous to within-person psychological conflict, and threatens the idea of an organism as an autonomous agent. “The Meaning of Life” refers to Haig’s interest in function and purpose, genetic information, and the origins of intentionality. These themes are also central in the work of Daniel Dennett, whose influence on Haig, and vice-versa, is apparent.

One striking feature of Haig’s book is the attention he pays to the *language* used in biology, in particular to the role of metaphors in conveying biological ideas. Indeed, in an Appendix in the book, Haig sketches the outline of a philosophy of language, which includes the striking claim that “all meaning is metaphor”. (I suspect that Haig does not realize how controversial this claim is among orthodox philosophers of language.) For a scientist steeped in molecular genetics and mathematical models of evolution, as Haig is, caring so much about the nuances of scientific language is rather unusual. Its source lies, I think, in Haig’s concern with the status of teleological (or apparently teleological) descriptors in biology, a perennial philosophical issue for evolutionists. Haig’s undergraduate educators, he reports, tried to instill the maxim “thou should not use teleological language”, a maxim that is still adhered to in some biological quarters today. Against this view, Haig believes that the “dogmatic exclusion of teleological considerations from the working philosophy of most biologists has become an impediment to scientific progress” (p. 373).

As is well-known, there are two different positions that scholars have taken on the issue of how Darwinism relates to teleology. The first is that Darwin banished teleology from biology by showing how a brute causal process—differential survival and reproduction—could eventually give rise to the appearance of design, purpose and goal-directedness in nature. The second is that Darwin *naturalized* teleology rather than eliminated it, by showing how purposive and teleological idioms can be translated into more respectable terms. Haig endorses the second of these positions, but with a twist. For unlike most proponents of the “naturalized teleology” position, his main interest does not lie in arguing that function, design and purpose are real features of the biological world (though he does think this). Rather, his concern is to understand how natural selection has given rise to “formal and final causes”, an Aristotelian-inspired category that Haig understands rather broadly, to include both information-bearing genes that lead to adaptive phenotypes and the deliberate actions of humans. As such, Haig is led to grapple with the notorious philosophical question of how meaning and intentionality can arise in a purely physical world, and how they can evolve by natural selection.

The parts of Haig’s book that I enjoyed the most were the ones that derive most directly from his scientific work, in particular his work on intra-genomic conflict. In the essay “Social Genes”, Haig recounts how Richard Dawkins’ second book, *The Extended Phenotype* (1982), that was published during his doctoral studies, transformed his worldview and led him to become a convinced gene selectionist. This revelation is unsurprising, since much of Haig’s subsequent work can be seen as pushing the logic of genic selection even further than Dawkins did, by uncovering the full extent of the genetic conflicts that can occur within a single organism and their remarkable phenotypic consequences. Haig follows Dawkins’ lead in treating an individual organism as akin to a social collective, comprised of genomic constituents with competing interests who do not necessarily “agree” on what their host organism should do. (Thus for example, paternally-derived alleles within a juvenile will favour a higher level of resource provisioning from their mother than will maternally-derived alleles.) Though familiar, this conceptualization of the organism still finds resistance from some critics who worry that it is too “geno-centric”. The essays in the first half of Haig’s book demonstrate the power and indeed near-inevitability of treating an organism as a genomic collective, drawing on a mix of theoretical considerations about the sorts of genetic conflict we should expect to find and empirical evidence about what we do actually find. Along the way, Haig makes an important conceptual advance by articulating his concept of a “strategic gene” and putting it to work.

The strategic gene concept is Haig’s attempt to clarify an issue that has long bedeviled gene selectionists, namely that of saying exactly what they mean by the term “gene”. Dawkins himself defined a gene as a length of DNA that is transmitted intact down the generations, i.e. that is not so long that it is likely to be broken up by crossing over at meiosis. This definition makes sense for Dawkins’ purposes (though Sterelny and Griffiths (1999) argue that Dawkins does not stick to it consistently) but it does not necessarily correspond to what molecular geneticists mean by a gene, as Dawkins himself noted. Other gene selectionists, such as Williams (1992), defined a gene in terms of information rather transmission, thus in effect treating a gene as an abstract entity. Haig’s starting point is to distinguish between material genes, i.e. the concrete DNA sequences that molecular biologists study, and informational genes, and to argue that the former stand to the latter as tokens to type, in philosophical parlance. (That is, many different material genes can all instantiate the same informational gene). However, he maintains that the informational gene is not quite the right understanding of the term “gene” for gene selectionists; and it is here that the strategic gene concept enters the picture.

A strategic gene, Haig tells us, is “a coterie of material genes” that interact because of recent common descent and “can be considered a unit of adaptive innovation”. To motivate this definition, Haig stresses that every genetic novelty, or informational gene, originates as a modification of an existing gene; thus material copies of this gene will interact with each other only when in different cells of the same body, or in bodies of closely related individuals. Only if the gene can spread under these conditions does it have a chance of becoming established. Thus the gene’s “strategy”, or phenotypic effect, must be such as to allow it to increase when rare. Haig notes that it is possible for a strategic gene to be coextensive with a material gene, if the gene acts in isolation from its other copies, but this is not the usual situation. In a multi-celled organism, for example, material genes expressed in somatic tissues promote the transmission of their copies in the organism’s germline; so the strategic gene becomes “a cluster of organism-sized genes”. And if a gene in the soma of one organism promotes the transmission of copies in relatives’ germlines (e.g. by causing altruistic behaviour), then the strategic gene is a “cluster of material genes distributed among some, but not all, members of a family.” Thus by attending to the interactions among the copies of a material gene (or more precisely, those interactions that influence transmission when copies of the gene are rare), Haig is able to provide a principled basis for determining which material copies belong in the coterie that constitutes a single strategic gene.

Though Haig does not explicitly say so, it is clear that his strategic gene is meant to be a concrete rather than an abstract entity, that is, it is a collective (rather than a set) composed of material genes that all instantiate the same informational gene and that interact because of recent common descent. I find that Haig’s three-way distinction between material, informational and strategic genes to be a major step forward, philosophically. The fact that gene selectionists have not always agreed on what they mean by “gene” has undoubtedly caused confusion among scientists, and despite extensive discussion, the attempts by philosophers to clarify the issue have not been entirely successful. I commend Haig’s careful treatment to anyone looking for a deeper understanding of this notorious issue.

Another chapter that stands out is “How Come? What For? Why?”, an extended version of a previously published essay in which Haig re-assesses Ernst Mayr’s famous distinction between proximate and ultimate causes. The chapter begins with some interesting historical material on how the notion of ultimate causation was understood in nineteenth century biology, then moves on to Mayr’s work, and then critiques some more recent contributions on the topic, including by philosophers of biology. Haig argues that the recent discussions of ultimate versus proximate involve much arguing at cross-purposes, since two distinctions have been conflated: between immediate and historical causes on the one hand, and between mechanism and adaptive function on the other. To see this conflation, notice that the question “how did it come to be so?” and “what is it for?”, are not equivalent (as Tinbergen emphasized); since depending on the case, the answer to the former question will not necessarily involve natural selection at all. According to Haig, Mayr himself identified ultimate causes with historical or temporally remote causes, and thus with “how did it come to be?” rather than “what for?”; but most of Mayr’s followers took him to be interested in the latter question, and thus understood proximate versus ultimate as a distinction between mechanism and adaptive function. Haig is surely correct that this is how Mayr’s followers interpreted him; and he makes a persuasive case for his claim that this is a misinterpretation, a point that I had not previously appreciated.

The essays in the final third of Haig’s book deal with meaning, interpretation, intentionality, consciousness, and free will. I found them to be interesting though hard going. In part, this reflects the intrinsic difficulty of the topics, and in part it is due to Haig’s rather freewheeling style and his penchant for grand philosophical pronouncements. (“The meaning of a life is the life that is lived. Your body is an interpretation of your life” (p. 314); “life is a cycle in which text and performance are reciprocally cause and effect of each other” (p. 363).) Statements such as these—which admittedly I have quoted out of context—may prompt the reader to worry that Haig has “gone over to the dark side” and fallen under the spell of continental philosophy, a worry made salient by the book’s title. But although Derrida does make a (mercifully brief) appearance in one chapter, this worry is largely misplaced. A fairer diagnosis, I think, is that Haig is trying to move over complex philosophical terrain too fast; and that he has not been aided by the inability of the (mostly analytic) philosophers that he has read to agree on exactly what they mean by terms such as “intentionality”, “interpretation” and “representation”, nor on what the right questions to ask are.

In conclusion, *From Darwin to Derrida* is a fascinating and erudite book that I strongly recommend. It is gratifying to find a scientist of Haig’s standing who takes philosophical questions so seriously, and who shows by example that dialogue between science and philosophy can yield rich dividends.
